# Polyamines as Snake Toxins and Their Probable Pharmacological Functions in Envenomation

**DOI:** 10.3390/toxins8100279

**Published:** 2016-09-26

**Authors:** Steven D. Aird, Alejandro Villar Briones, Michael C. Roy, Alexander S. Mikheyev

**Affiliations:** 1 Division of Faculty Affairs, Okinawa Institute of Science and Technology Graduate University, 1919-1 Tancha, Onna-son, Kunigami-gun, Okinawa-ken 904-0495, Japan; steven.aird@oist.jp; 2 Ecology and Evolution Unit, Okinawa Institute of Science and Technology Graduate University, 1919-1 Tancha, Onna-son, Kunigami-gun, Okinawa-ken 904-0495, Japan; 3 Division of Research Support, Okinawa Institute of Science and Technology Graduate University, 1919-1 Tancha, Onna-son, Kunigami-gun, Okinawa-ken 904-0495, Japan; avillar@oist.jp (A.V.B.); mcroy@oist.jp (M.C.R.)

**Keywords:** polyamines, snake venoms, spermine, spermidine, putrescine, cadaverine, titer, pharmacology, envenomation sequelae

## Abstract

While decades of research have focused on snake venom proteins, far less attention has been paid to small organic venom constituents. Using mostly pooled samples, we surveyed 31 venoms (six elapid, six viperid, and 19 crotalid) for spermine, spermidine, putrescine, and cadaverine. Most venoms contained all four polyamines, although some in essentially trace quantities. Spermine is a potentially significant component of many viperid and crotalid venoms (≤0.16% by mass, or 7.9 µmol/g); however, it is almost completely absent from elapid venoms assayed. All elapid venoms contained larger molar quantities of putrescine and cadaverine than spermine, but still at levels that are likely to be biologically insignificant. As with venom purines, polyamines impact numerous physiological targets in ways that are consistent with the objectives of prey envenomation, prey immobilization via hypotension and paralysis. Most venoms probably do not contain sufficient quantities of polyamines to induce systemic effects in prey; however, local effects seem probable. A review of the pharmacological literature suggests that spermine could contribute to prey hypotension and paralysis by interacting with *N*-methyl-d-aspartate (NMDA) and α-amino-3-hydroxy-5-methyl-4-isoxazolepropionic acid (AMPA) receptors, nicotinic and muscarinic acetylcholine receptors, γ-Aminobutyric acid (GABA) receptors, blood platelets, ryanodine receptors, and Ca^2+^-ATPase. It also blocks many types of cation-permeable channels by interacting with negatively charged amino acid residues in the channel mouths. The site of envenomation probably determines which physiological targets assume the greatest importance; however, venom-induced liberation of endogenous, intracellular stores of polyamines could potentially have systemic implications and may contribute significantly to envenomation sequelae.

## 1. Introduction

In an ongoing investigation of protein expression regulatory networks in venom glands of the Taiwan habu (*Protobothrops mucrosquamatus*), we were surprised to discover that the gene for spermine synthase was strongly upregulated, along with genes for well-established venom proteins. This indicated that spermine has some role either in the venom synthetic machinery or is a constituent of the venom itself. A search of the snake venom literature revealed only three papers that mentioned spermine. In 1960, using paper chromatography, Sasaki [1] identified spermine and histamine in the venom of the Taiwan habu (*Protobothrops mucrosquamatus*). In 1965, Shipolini et al. [2] found that the venom of the Bulgarian viper (*Vipera ammodytes ammodytes*) contains spermine, but not histamine. No further reports of spermine appeared in the literature until 2007, when Merkel et al. identified spermine as the trypanicidal component in the venom of the viper, *Eristocophis macmahoni* [3]. While none of the foregoing papers addressed the question of spermine’s function in envenomation, Merkel et al. reported that it constituted 1% of the dry mass of their venom sample. This high titer suggested that spermine has a functional role in envenomation, and that it is not simply a fortuitous trypanicide.

**Figure 1:**
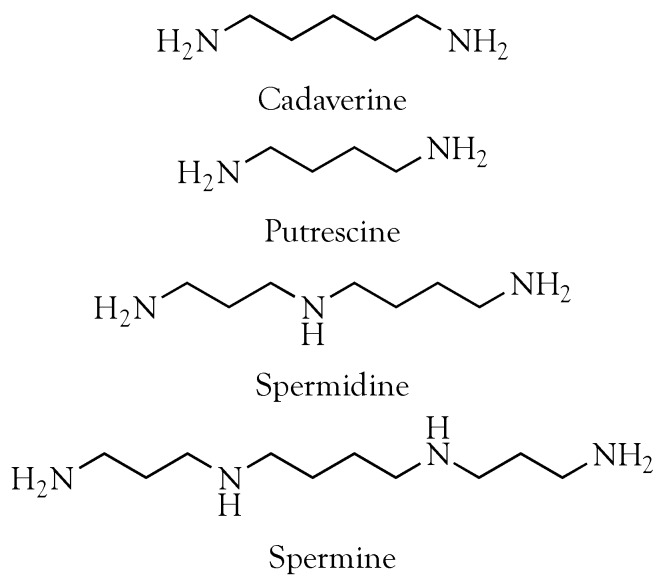
Structures of putrescine, cadaverine, spermidine, and spermine. At physiological pH, the amino groups are positively charged.

Unlike the arylpolyamines that are abundant components of many spider venoms [4,5,6,7,8], the structures of putrescine, cadaverine, spermidine, and spermine effectively render them invisible at 280 nm (Figure 1), traditionally used in liquid chromatography of snake venom proteins, because that wavelength detects the aromatic amino acids, tryptophan, tyrosine, and phenylalanine. The long-UV invisibility of polyamines, coupled with their small size, probably explains their virtual absence in the snake venom literature.

Because most snakes prey upon mammals, we used a combination of polyamine derivatization and liquid chromatography-mass spectrometry to identify and quantify the four mammalian polyamines spermine, spermidine, putrescine, and cadaverine, not only in the venom of *Protobothrops mucrosquamatus*, but also in a selection of Old and New World elapid, viperid, and crotalid venoms. Here we confirm their presence in a phylogenetically diverse array of snake venoms. All polyamines display broad taxonomic distributions. The abundance of spermine confirms that it is a significant constituent of many viperid and crotalid snake venoms and implies a functional role in envenomation. Here we present the results of our polyamine taxonomic survey. We review the pharmacology of polyamines that appears pertinent to snake envenomation and we discuss the possible contributions of spermine and other polyamines to prey immobilization.

## 2. Results

### 2.1. Detection, Quantification, and Distribution of Polyamines in Snake Venoms

We assayed 31 venoms representing the three major lineages of advanced venomous snakes (Elapidae, Viperinae, and Crotalinae) (Table 1) by LC-MS to determine whether they contained spermine, and if so, to quantify how much was present. In the process we unexpectedly also identified putrescine, spermidine, and cadaverine in all venoms (Figure 1). In fact, most venoms examined contained all four polyamines, but quantities of each varied dramatically among taxa (Table 1).

**Figure 2:**
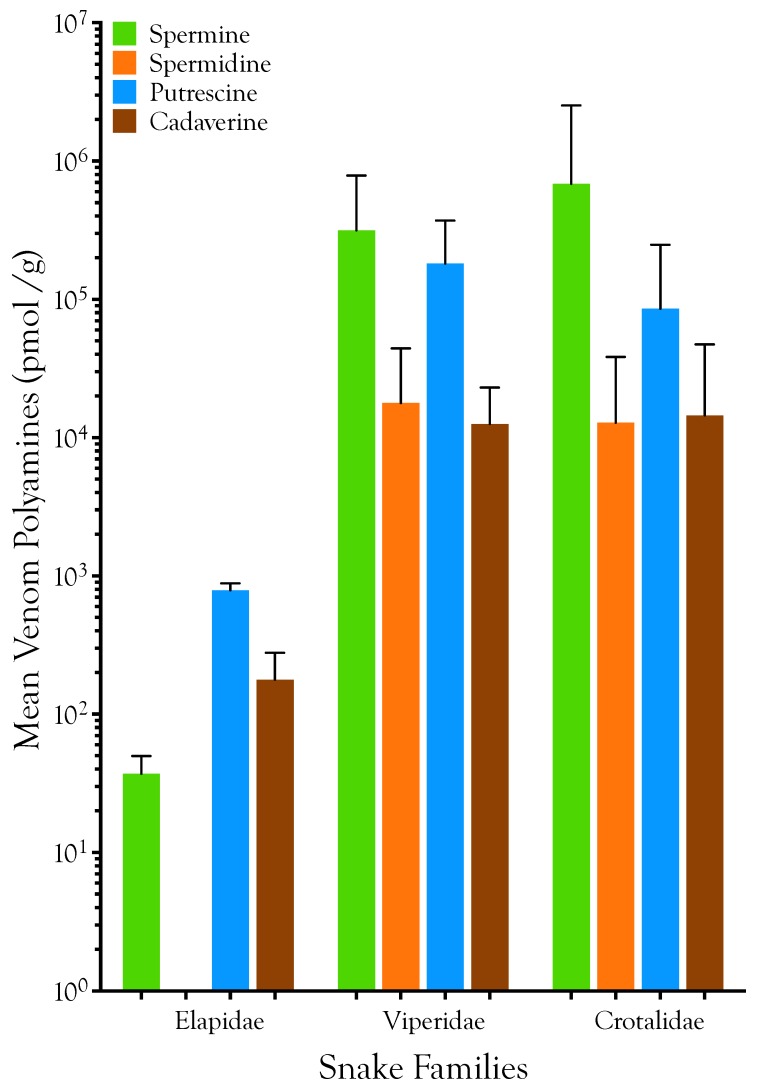
Polyamines are apparently components of the venoms of all advanced venomous snakes. They are extremely minor constituents of elapid venoms, and venom polyamines probably contribute little to elapid envenomations. Putrescine is the dominant elapid venom polyamine. Viperid and crotalid venom polyamines are much more abundant, but whether they are able to induce significant systemic physiological impairment in prey, independent of other venom constituents, is unclear. However, it is possible that they provoke localized responses, such as skeletal muscle paralysis at the site of injection. Spermine and putrescine are the most significant viperid and crotalid polyamines, but variation between taxa is large. Because polyamine abundance spans several orders of magnitude, bars show the log_10_ of polyamine concentration in pmol/g venom. Error bars indicate 1 standard deviation.

**Figure 3:**
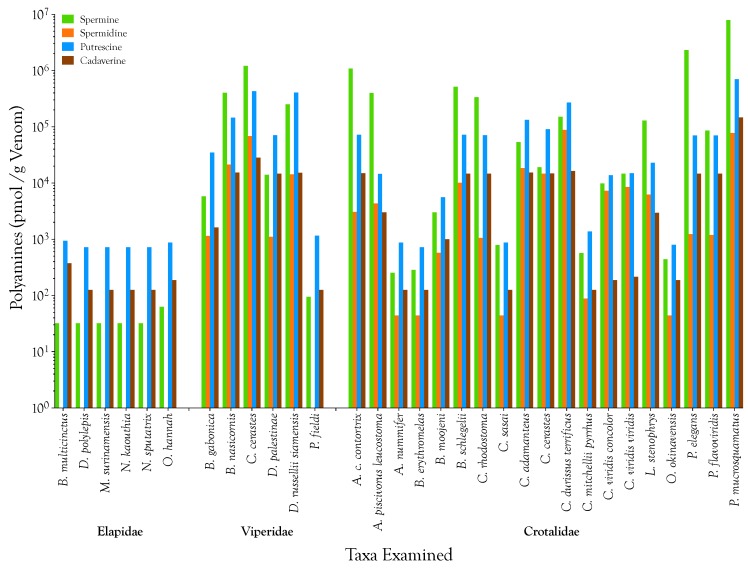
Polyamine levels in venoms of 31 venomous snake taxa, grouped by family and arranged alphabetically within families. The range of polyamine spanned several orders of magnitude, necessitating a logarithmic Y axis. For that reason, values are expressed as pmol polyamine/g venom. Polyamines are negligible constituents of the elapid venoms examined. They are relatively more abundant in viperid and crotalid venoms, but beyond that it is difficult to discern any distributional patterns at higher taxonomic levels. Interspecific variability, within genera, is considerable, as shown for the genera *Bitis*, *Bothrops*, *Crotalus*, and *Protobothrops*. Spermine is generally the most abundant polyamine, although in the elapid venoms examined and in our samples of *Bitis gabonica*, *Daboia russellii siamensis*, *Crotalus durissus terrificus*, *Crotalus mitchellii pyrrhus*, and *Crotalus viridis concolor* venoms, putrescine was dominant. Spermidine was a significant component only in the venoms of two *Crotalus viridis* subspecies, from geographically contiguous regions in western Colorado. Significant levels of cadaverine were seen only in *Protobothrops mucrosquamatus* venom.

Polyamines are relatively minor components of elapid venoms (Figure 2 and Figure 3; Table 1) and it is doubtful that they serve significant functions in envenomation. Specifically, spermine is almost absent from the six elapid venoms we examined (mean = 0.007 µg/g; range 0.006–0.013 µg/g; *n* = 6 species; pooled samples). Even though it is an intermediate in the spermine anabolic pathway, spermidine is universally absent in elapid venoms. In contradistinction to most viperid and crotalid venoms, putrescine (mean = 0.069 µg/g; 0.064–0.083 µg/g) and cadaverine (mean = 0.018 µg/g; 0.013–0.038 µg/g) are the dominant polyamines, although in elapid venoms these are still extremely minor venom components at best.

Spermine is relatively much more abundant in viperid venoms (µ = 63.8 µg/g; range 0.019–247 µg/g; *n* = 6 species; pooled and individual samples), and reaches its highest concentrations in some crotalid venoms (µ = 139 µg/g; 0.051–1600 µg/g; *n* = 19 species; pooled and individual samples). In *Protobothrops mucrosquamatus* venom, ~7.9 µmol spermine is present per gram of venom (Figure 2 and Figure 3; Table 1).

While spermine is the major polyamine in most venoms examined, putrescine was the most abundant polyamine in all six elapid venoms and in those of *Bitis gabonica*, *Daboia palestinae*, *Pseudocerastes fieldi*, *Atropoides nummifer*, *Bothrops erythromelas*, *Crotalus adamanteus*, *Crotalus cerastes*, and *C. mitchellii pyrrhus* (Figure 3; Table 1). Spermidine was a major polyamine only in venoms of Crotalus durissus terrificus and Protobothrops mucrosquamatus. Cadaverine, which is produced from lysine via a different anabolic pathway than the other three polyamines, was a minor to negligible constituent in all venoms examined (Figure 3). It reached its highest levels in the venoms of Protobothrops mucrosquamatus (Figure 3; Table 1).

Merkel et al. reported a value of 9.14 µg/mg (9.14 mg/g) of spermine in the venom of the viperid, Eristocophis macmahoni [3]. This is nearly 6× higher than the highest value recorded in our study (Protobothrops mucrosquamatus) and 37× higher than the highest viperid venom (Bitis gabonica). Merkel et al. have clearly identified spermine in Eristocophis venom; however, we believe that their quantification is spuriously high. Given that their calibration curves look appropriate, several factors may contribute to this discrepancy. First, they did not derivatize their sample, in which case, various polyamines could produce the same fragments [9]. However, the sum of all other polyamines is unlikely to be large enough to account for the difference between their value and ours. In our initial experiments, we did not derivatize the polyamines either. We found that the spermine standard eluted from the C18 column with poor reproducibility. This problem can be exacerbated by other polyamines or other classes of compounds present in venom. Chromatographic methods to circumnavigate this difficulty without derivatization tend to be fairly complex, in part because ion pairing reagents often used to improve chromatographic separations result in signal suppression for basic compounds during mass spectrometry [10]. For this reason, we opted to derivatize our samples. Some derivatization methods in the literature do not go to completion, resulting in multitudes of partial derivatives and rendering quantification impossible. However, the method of Liu et al. [11] yields a single, detectable peak with each of the polyamines assayed. Liu et al. reported a significant increase in sensitivity based upon derivatization with benzoyl chloride; thus our values for spermine ought to have been higher than those of Merkel et al. We cannot explain the difference.

**Figure 4:**
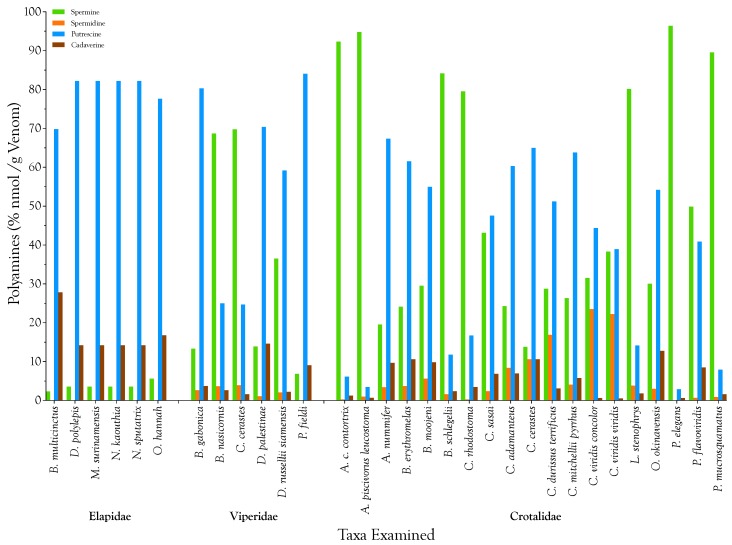
With regard to polyamine composition, venomous snake taxa employ either a putrescine- or a spermine-dominant strategy. Polyamine concentrations in each of 31 venomous snake taxa were normalized by expressing them as percentages of total nmol of polyamine for the taxon (sample) in question; thus all the polyamines in a given sample sum to 100%.

While it is probably meaningless to speak of “polyamine strategies” in relation to elapid venoms, for those species employing significant polyamine titers in their venoms, it is interesting to see how different species have allocated their resources with regard to the polyamine composition of their venoms. For this purpose, total nmol of polyamine per venom sample were calculated. Then each of the four polyamine concentrations was normalized by expressing it as a percentage of the total for that venom sample (Figure 4). All venoms adopted either a putrescine- or spermine-dominant strategy, sometimes to the near exclusion of all other polyamines (*Agkistrodon c. contortrix*, *A. piscivorus leucostoma*, *Protobothrops elegans*, and *P. mucrosquamatus*) (Figure 4).

**Figure 5:**
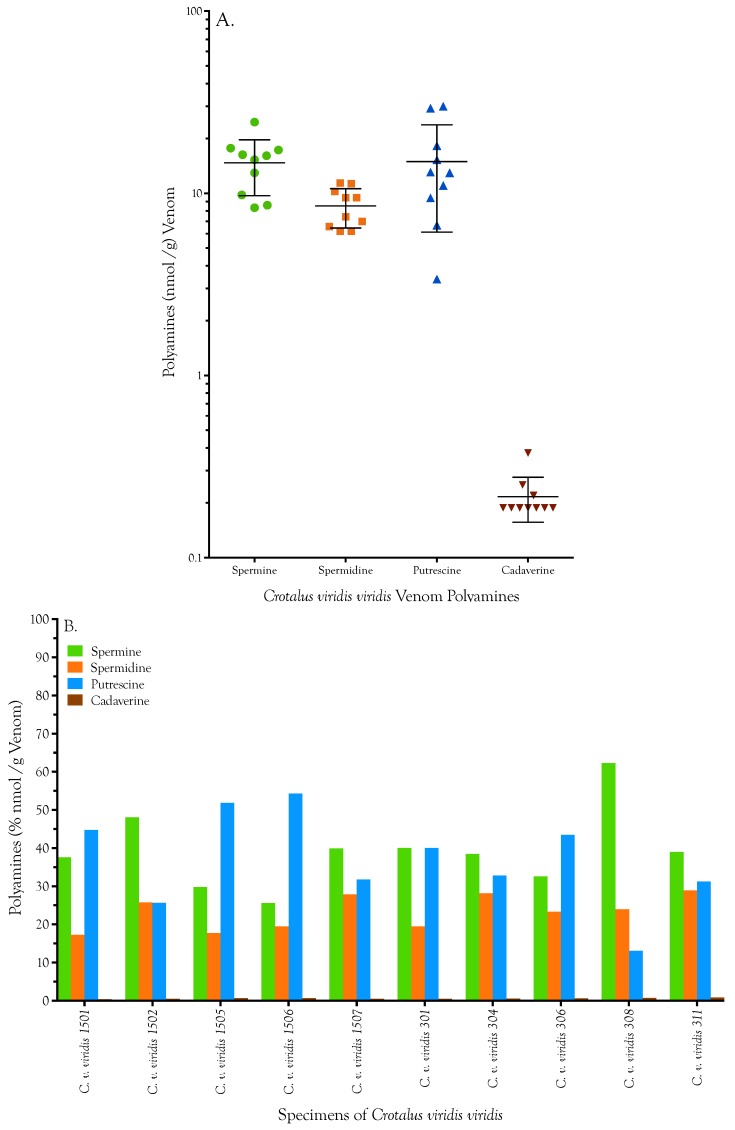
Polyamine titers in the venoms of five *Crotalus viridis viridis* from each of two populations in western Colorado (Buford, Rio Blanco County, CO and Brown’s Park, Moffat County, CO, USA) (nmol polyamine/g venom). (**A**) Spermine and putrescine showed the highest titers and the greatest dispersion of values. Means for each polyamine are shown ± one standard deviation. (**B**) Interestingly, in all 10 specimens, on a mass basis, spermine was the most abundant polyamine, while cadaverine was least. However, on a molar basis, spermine was most abundant only in five specimens, while putrescine was most abundant in four.

In order to assess the amount of intraspecific variation in polyamine titers, venoms from 10 specimens of Crotalus viridis viridis, representing two western Colorado populations (Buford, Rio Blanco County and Brown’s Park, Moffat County) (Figure 5, Table A1). Two replicates of each specimen were run for each polyamine. We examined the amount of run-to-run variability and intraspecific variation using a multivariate linear model of polyamine concentrations. There was no significant effect of technical replication (*p* = 0.94), and a small effect of population (*p* = 0.045). However, the variation between populations was much smaller than that between species (Bartlett’s test *p*-value < 2.2 × 10−16 in all cases). Replicates of each specimen were highly consistent for all specimens and for all four polyamines (Figure 5, Table A1). In terms of mass, spermine was the most abundant polyamine in all 10 C. v. viridis venoms, while cadaverine was least abundant. However, when considered on a molar basis, the picture changed, except that cadaverine was still least abundant (Figure 5b). On a molar basis, spermine predominated in five specimens, while putrescine was dominant in four. In one specimen they were co-equal.

### 2.2. Biological Significance of Snake Venom Spermine

Prey classes consumed were documented for 28 of 31 species (Table A2). Personal observations were available for *Protobothrops elegans* and prey were inferred for the remaining two species, based upon the diets of congeners. Using the R statistical package [12], we fit a multivariate linear model with patterns of polyamine abundance vs. prey consumed by the snake species examined to determine whether the polyamine compositions are related to prey species eaten. However, there was no effect of any prey species on polyamine concentration (*p* > 0.1 in all cases). There are several possible explanations for this result. We could test only for prey taxa that have been documented. Because such data are not abundant in the literature, prey data compiled here are likely incomplete. That is, many species probably eat other prey types not recorded here. On the other hand, it may not make any difference. Polyamines are clearly minor venom components in many species, and in those species, polyamine abundance is likely to be under minimal selective pressure [13]. Alternatively, there is clearly significant polyamine variation at the family, and perhaps even at the generic level (Figure 4), which could imply the existence of strong phylogenetic constraint acting on these components.

At this point, we cannot categorically exclude the possibility that venom polyamines serve some function in the gland, either to stabilize or temporarily inactivate venom constituents, or to act in a direct regulatory manner on glandular tissue. However, given the phylogenetic patterns mentioned above, we think such explanations for their presence are unlikely.

#### Quantities of Polyamines Potentially Injected

Hayes [14] reported that prairie rattlesnakes (*Crotalus viridis*) injected an average of 16 mg of venom when striking mice. In cases involving second bites on the same mouse, that value increased to 22.4 mg, but multiple bites accounted for only about 14% of all feeding strikes. Morrison et al. [15] found that tiger snakes (*Notechis scutatus*) injected 12.7 mg on average, while taipans (*Oxyuranus scutellatus*) injected 20.8 mg. Subsequently they reported data for a variety of additional elapids [16]. Death adders (*Acanthophis antarcticus*), which resemble small viperids, delivered an average of 30.7 mg, while the much larger king brown snakes (*Pseudechis australis*) averaged 61.6 mg. Other elapids (*Pseudonaja textilis*, *Tropidechis carinatus*, *Pseudechis collettii*, and *P. porphyriacus*) injected much smaller quantities.

Other factors that influence the amount of venom injected include the temperament and strike behavior of the species, the size of the snake (intraspecific comparisons only), the species and behavior of the prey, and the quantity of venom that the species possesses when the glands are filled to capacity. In regard to the latter variable, over the course of thousands of venom extractions from adult rattlesnakes and other pit vipers, the first author routinely obtained yields of 250–400 µL per extraction (60–140 mg) and one 75-cm *Agkistrodon piscivorus* once produced an astonishing 1.1 mL (275–385 mg).

If we assume that adult viperids and crotalids are capable of injecting 10–50 mg of venom, using the lowest and highest polyamine titers registered in this study (1.18–8843 nmol/g) (Table 1) we can predict that injections of total polyamines would be in the range of 0.01–442 nmol per injection, or 0.005–221 nmol per fang puncture. For the sake of comparisons, the spermine content of normal erythrocytes is approximately 6–9 nmol/10^10^ erythrocytes [17,18].

Tabor and Rosenthal [19] reported a transient 26% drop in blood pressure (115 mm Hg to 85 mm) in conscious rats injected i.v. with 0.15 µmol/g of spermine. The response was of the same magnitude as that induced by comparable doses of histamine. The same dose caused 83% mortality in mice due to nephrotoxicity, regardless of injection route (LD_50_ = 0.128 µmol/g; 2560 nmol/20 g mouse); however, i.v. doses given too rapidly caused immediate death. None of the species examined in this study appear to be capable of injecting quantities of this magnitude into their prey (Table 1).

On the other hand, a snake bite certainly constitutes a rapid injection of a dose, and intravascular punctures undoubtedly occur during predatory strikes. Polyamine oxidation results in the liberation of ammonia, hydrogen peroxide, and acrolein [20,21,22]. Acrolein is highly cytotoxic, but the time course is likely too long to be relevant to envenomation. While small quantities of hydrogen peroxide can probably be metabolized by erythrocyte catalase, oxidation of bolus doses of polyamines probably results in the liberation of lethal quantities of ammonia and peroxide. Moreover, many crotalid venoms contain approximately 100 peptidyl constituents, many of which have pharmacological effects that overlap those of the four polyamines investigated [13,23]. On numerous occasions, the senior author has observed mice bitten by captive rattlesnakes that rolled over or collapsed without taking a single step. Paralysis was effectively instantaneous.

Except in cases involving *Cerastes cerastes*, *Agkistrodon contortrix contortrix*, *Protobothrops elegans*, and *P. mucrosquamatus*, venom polyamines may exert effects restricted to the bite locale, rather than systemic effects (Figure 2; Table 1). Nonetheless, as will be evident from the discussion of polyamine pharmacology pertinent to envenomation that follows, those local effects should probably not be discounted.

In addition, liberation of endogenous, intracellular polyamine stores from prey tissues, especially leukocytes and erythrocytes [17], by other venom components, could result in a potentially significant contribution to envenomation sequelae, a phenomenon that has been suggested for other types of tissue injury [24]. Polyamine concentrations vary widely between tissue types (from as little as 202 nmol/g of spermine and 145 nmol spermidine in mouse muscle to 5708 nmol/g spermine and 7725 nmol spermidine in the rat prostate) [25]. Comparable values (hundreds of nmoles) have been reported for spermidine in guinea pig tissues [26] and spermine and spermidine in rabbit central and peripheral nervous tissues [27] and the cat brain [28].

Paschen [29] suggested four different mechanisms of polyamine-dependent cell injury. Two of those appear especially pertinent to envenomation: an overactivation of calcium flux resulting in neurotransmitter release and an overactivation of the NMDA receptor complex. Nonetheless, polyamines have various physiological impacts that may contribute to prey immobilization. A brief review of some of these mechanisms is provided below.

### 2.3. Pharmacology of Spermine that Is Potentially Pertinent to Envenomation

Despite its name, spermine is a constituent of all eukaryotic cells [30,31] and it occurs in vertebrate blood plasma at µM levels [32]. It is also found in synaptic vesicles at concentrations as high as 2.8 mM [33]. Spermine is co-secreted with neurotransmitters [34] and polyamine transport systems exist in glial cells and synaptosomes [33].

Spermine has numerous pharmacological functions that are relevant to snake envenomation of vertebrate and invertebrate prey. Because it is ubiquitous in animal tissues [30,31], like purine nucleosides [35,36], its inclusion in venom represents something of a trump card in the predator-prey arms race. No prey species can possibly develop resistance to it.

#### 2.3.1. Spermine Promotes Hypotension

The acylpolyamines, putrescine, spermidine, and spermine, are constituents of nearly all cells [37]. At physiologic pH, spermine carries a +4 charge and can react ionically with nucleic acids and negatively charged regions of proteins. For this reason, spermine and other polyamines function as Ca^2+^ antagonists [38,39,40]. De Meis [41] was the first to report that spermine (3 mM) relaxed guinea pig ileum and taenia coli precontracted with acetylcholine, histamine, nicotine, caffeine, or excess potassium. It also relaxed frog skeletal muscle precontracted with acetylcholine, 28 mM potassium, or electrical stimulation. Hashimoto et al. [39] found that 400 µM spermine inhibited spontaneous contractions of rat uterus, an effect that could be overcome by increasing extracellular Ca^2+^ concentrations. Intravenous injections of spermidine and spermine also induced significant cardiovascular changes in anaesthetized dogs, albeit with lower potencies than catecholamines [42]. Marmo et al. [42] concluded that the hypotensive response was due to histamine release and that i.v. injections of spermine in the vertebral artery elicited hypotensive response via an increase in parasympathetic output. Spermine also decreased baroreceptor reactivity.

Chideckel et al. [43,44] found that polyamines (2 mg/kg) (calculated < 10 µM) relax uterine, gastrointestinal, and respiratory tract smooth muscle, and cause hypotension by reducing peripheral vascular resistance, when infused into rats or dogs. Vasodilation was unaffected by histamine H1 or H2 antagonists, a result that contradicted the conclusion of Marmo et al. Fernández et al. [45] concluded that polyamines inhibit smooth muscle contraction by acting at the plasma membrane to reduce calcium influx. Nilsson et al. [46] reported that 100 µM spermine reduced contractile activity of rat portal vein, and that 1 mM spermine abolished it. Similar results have been obtained using rat bladder strips (10–100 µM spermine) [47] or rat uterus [48]. These effects are evidently mediated by blockade of L-type Ca^2+^ channels in vascular smooth muscle [49].

Vertebrate arterial endothelial cells express Ca^2+^-sensing receptors (CaR) [50]. In human embryonic kidney cells (HEK-293) CaRs are activated by increases in spermine concentration [51]. The resulting influx of extracellular Ca^2+^ liberates IP_3_- and ryanodine-sensitive intracellular Ca^2+^ stores [52], leading to the production of nitric oxide [53], and activating IK_Ca_ channels [50], resulting in vasodilation.

Spermine also affects cardiac muscle. The negative inotropic effects of 100–500 µM spermine, reported by Ventura et al. [54] are not mediated by nitric oxide or histamine [55]. Guevara-Balcazar et al. found that a P2Y purine receptor antagonist and a nonspecific adenosine receptor antagonist blocked the effects of spermine and concluded that the negative inotropy is mediated at least in part by ATP release.

#### 2.3.2. Spermine Blocks L-Type Voltage-Dependent Calcium Channels, but Exerts Biphasic Effects on N-Type Channels

Six types of voltage-dependent calcium channels (VDCCs), known as *L*-, *N*-, *P*-, *Q*-, *R*-, and *T*-type channels, have been characterized to date [56,57]. When VDCCs open, Ca^2+^, which is 2 × 10^4^ times more abundant in the extracellular fluid than in the cytoplasm, rushes into the cell [58]. *N*- (Ca_V_2.2), *P*-, and *Q*-type channels (Ca_V_2.1) are primarily responsible for neurotransmitter release at various central sites. R-type channels (Ca_V_2.3) may play a minor role in some cases, but apparently serve primarily to govern synaptic plasticity [56]. It is widely thought that *L*- (Ca_V_1.1-1.4) and *T*-type channels (Ca_V_3.1-3.3) generally do not participate in excitatory neurotransmitter release [56,59]. They are present in cardiac and smooth muscle cells and in many neuronal cells. Both open in response to depolarizing stimuli, but *T*-type channels open at more negative membrane potentials and do so transiently. *L*-type channels open for longer periods; hence the name. We are unaware of any studies of interactions between spermine and *R*-type or *P*-type channels. Herman et al. [60] reported that spermine had no effect on *T*- or *L*-type VDCCs in mouse neuroblastoma cells. Eterovic et al. [61] opined that 40% of the calcium channels in rat hippocampal slices were probably of the *Q*-type, but there do not appear to have been any confirming studies.

One of the earliest reports regarding the action of spermine on VDCCs was that of Pullan et al. [62], who found that spermine competes with ω-conotoxin GVIA at presynaptic *N*-type VDCCs. Spermine exerts biphasic effects on *N*-type Ca^2+^ channels (CaV2.2), which are involved in neurotransmission at many fast synapses, facilitating Ca^2+^ entry into neuronal cell bodies at nM concentrations, but inhibiting it at µM levels [34].

*L*-type channels are well known for their role in contraction of smooth (Ca_V_1.2), cardiac (Ca_V_1.1-1.2), and skeletal muscle (Ca_V_1.1), but they also govern liberation of catecholamines from chromaffin cells, dynorphin from rat hippocampal granule cell dendrites, neuropeptides from the posterior pituitary gland, and in at least some cases, excitatory neurotransmitters (e.g., retina) [57,59]. Using the whole-cell patch clamp technique, Bonci et al. reported that *L*-type channels on presumed dopaminergic neurons of rat midbrain activated NMDA and metabotropic glutamate receptors, via the probable release of aspartate and glutamate.

Schoemaker [63] also concluded that endogenous polyamines function as modulators of *L*-type VDCCs. Gomez et al. [49] found that spermine inhibited contractions of intestinal smooth muscle in a dose-dependent manner with an EC_50_ of about 1 mM. Since spermine blocked current induced by 1 µM BAY K 8644, but could not enhance the inhibition caused by 1 µM nifedipine, they concluded that spermine acts by inhibiting Ca^2+^ currents responsible for generation of action potentials. DiScenna et al. [24] opined that spermine’s reduction of evoked potentials at AMPARs and GABAA receptors is best explained by a blockade of VDCCs, and Eterovic et al. [54] went so far as to suggest that all spermine effects can be explained by inhibition of VDCCs. This conclusion was later supported by Joshi et al. [64] who concluded that in motor neurons, a small portion of the Ca^2+^ current associated with α-amino-3-hydroxy-5-methyl-4-isoxazolepropionic acid (AMPA) receptors is attributable to Ca^2+^-permeable (Type II) AMPA receptors; however, the majority of it is carried by *L*-type Ca^2+^ channels activated by AMPA receptors. Cayzac et al. [65] reported that 300 µM spermine inhibited *L*-type Ca^2+^ (Ca_V_1.2) channels heterologously expressed in HEK 293 cells, but that 100 nM spermine had no effect, suggesting that at least, Ca_V_1.2 channels do not manifest the biphasic spermine response displayed by N-type channels.

#### 2.3.3. Spermine and Calcium Release (Ryanodine) Receptors

Ryanodine receptors are intracellular Ca^2+^ receptors that operate by a positive feedback mechanism. When they bind Ca^2+^ on their cytoplasmic surfaces they depolarize the sarcolemma, releasing far more Ca^2+^ from sarcoplasmic reticulum and other intracellular Ca^2+^ stores, resulting in muscle contraction [66]. The mechanisms by which muscle contraction ensues vary between muscle types. Uehara et al. [67] concluded that spermine and other polyamines block the RyR channel, thereby diminishing sarcoplasmic reticulum Ca^2+^ release. Zarka and Shoshan-Barmatz [68] specifically reported that spermine stimulated binding of ryanodine, an inhibitor of ryanodine receptors, by up to 5-fold, with a concentration of 3.5 mM spermine producing a half-maximal effect.

#### 2.3.4. Spermine Potentiates NMDA Receptors (NMDARs) at µM Concentrations, but Inhibits Them at mM Concentrations

The *N*-methyl-d-aspartate receptor (NMDAR) contains an integral ion channel that permits entry of Na^+^, K^+^, and Ca^2+^, but is blocked by Mg^2+^ [69]. Excessive stimulation of NMDARs increases intracellular Ca^2+^, resulting in excitotoxicity [70]. Responses of *Xenopus* oocytes expressing whole rat brain or chick cerebellar NMDARs to NMDA and kainate were potentiated by 10–100 µM spermine, suggesting a polyamine binding site on the NMDAR [71]. McGurk et al. likewise found that spermine potentiated responses of NMDAR in the presence of glycine [72]. The latter two studies reported that higher spermine concentrations inhibited responses to glutamate and aspartate, implying different mechanisms of action for excitation and inhibition, a result confirmed by later investigations [73].

Calcium influx is thought to be responsible for the role of NMDARs in long-term depression and long-term potentiation, relevant to learning and memory [74]; however, pre-synaptic NMDARs also modulate synaptic transmission in the spinal cord [75], and NMDARs are responsible for part of the motor drive to respiratory muscles [76,77,78]. More importantly, in all vertebrates from lampreys to primates, NMDARs in the brainstem and spinal cord are involved in coordination of locomotion, regardless of the type of locomotion [79,80].

The polyamine binding site on NMDARs is distinct from the binding sites for glutamate, glycine, Mg^2+^, Zn^2+^, and open-channel blockers such as MK-801 [81,82]. Marvizón and Baudry also found that spermine (1 µM–1 mM) increased MK-801 binding to NMDARs in the presence of glutamate and the glycine antagonists, 7-chlorokynurenate (10 µM) or DNQX (10 µM), which glutamate alone does not do. Pullan and Powel [83] showed that 100 µM spermine reduced the binding constant of L-glutamate by about half.

Benveniste and Mayer [84] confirmed that spermine acts at multiple sites on NMDA receptors from rat hippocampal neurons, but also found that spermine exhibits biphasic effects and that it can either potentiate or block ion permeability. They further reported that potentiation results from both an increase in NMDAR affinity for glycine (EC_50_ = 125 µM) and in an increase in the maximum response to NMDA when NMDARs are saturated with glycine. They concluded that NMDAR blockade is voltage-dependent, and that it is due to polyamine binding to sites inside the ion channel. Robichaud and Boxer [85] found that spermine increased the frequency of spontaneous discharges from rat neocortical slices in Mg^2+^-free buffer at concentrations below 1 mM, exhibited biphasic effects at 1 mM, and reduced discharge frequency at 3 mM. However, discharge amplitude only diminished in a concentration-dependent manner with spermine concentrations in the range of 300 µM to 3 mM. Araneda et al. [86] also reported biphasic effects of spermine. They found that 300 µM spermine approximately doubled the response to 30 µM NMDA in the presence of 1 µM glycine. In rat hippocampal neurons, extracellular spermine potentiated NMDAR currents by increasing the open channel probability at membrane potentials ranging from −70 to +40 mV (EC_50_ = 215 µM). They suggested that the channel block at higher spermine concentrations might be due to binding of negatively charged residues in the channel entrance by spermine’s amino groups. Ferchmin et al. [87] reported that spermine is excitotoxic at µM concentrations because it activates NMDARs [88,89], but it neuroprotective at mM concentrations because it blocks the receptor ion channel. Turecek et al. [90] proposed that 10 mM intracellular spermine directly inhibits NMDA receptors in a manner that is different from calcium-induced NMDA receptor inactivation and spermine-induced voltage-dependent inhibition of AMPA/kainate receptors.

Calderon and Lopez-Colome [91] found that spermine inhibited specific [^3^H]glycine binding to membranes from synaptosomal fractions from the inner (IC_50_ = 32 µM) and outer (IC_50_ = 35 µM) plexiform layers of 1–3 day-old chick retinas in a dose-dependent manner. They concluded that their results demonstrate a single spermine binding site on NMDARs in both layers of the chick retina. Mortensen et al. [92] found that spermine binding sites on NMDARs in four human cortical regions showed regional and individual variation in their capacity to enhance binding of the NMDAR open channel blocker, MK-801. Ragnarsson et al. [93] suggested that tissue differences in NMDAR susceptibility to modulation by spermine may result from different NMDAR subunit compositions.

Doyle and Shaw [94] found that 100-µg injections of spermine into the left lateral cerebral ventricle of mice produced two very different phases of CNS excitatory effects. The first, of rapid onset, involved scratching and frequent face washing, while some mice developed clonic convulsions. The second phase involved body tremors that intensified until they climaxed in fatal tonic convulsions. The authors concluded that while the two phases were pharmacologically quite different, both were probably mediated by NMDARs. Kirby and Shaw [95] reported that by virtue of its action at NMDARs, 300 µM spermine causes an increase in the rate of spontaneous epileptiform discharges in epilepsy-prone mice.

Adriani et al. [96] reported that, in mice, NMDA receptor blockade stimulated locomotor activity in a manner similar to MK-801 or dopamine agonists; however, low doses of NMDA or dopamine antagonists impair murine reactivity to spatial changes. Disorientation, rather than stimulation of locomotion would be strategically consonant with the objectives of envenomation, and would be consistent with the locomotor suppression induced by purine nucleosides via central A_1_ and A_2_ adenosine receptors [35,97,98,99,100]. Choi [66] opined that NMDA receptor activation might trigger neuronal death more rapidly than AMPA or kainate receptor activation, possibly due to a greater capacity to induce calcium influx and subsequent cytoplasmic calcium concentration.

Hardingham and Bading [101] report that neuronal NMDARs comprise two populations: synaptic receptors, which are neuroprotective, and extrasynaptic receptors that promote excitotoxicity and neuronal death. While the latter diminish during development, they can comprise 75% of all NMDARs. The normal physiological role of extrasynaptic NMDARs is poorly understood. Choi reported that the NMDAR is the primary site for toxic Ca^2+^ influx [102] and Tymianski et al. found that Ca^2+^ influx through NMDARs is more effective at inducing neuronal death than that through voltage-gated Ca^2+^ channels [103].

#### 2.3.5. Spermine Blocks Type II AMPA Receptors (AMPARs)

AMPARs are responsible for fast, excitatory neurotransmission in the vertebrate central nervous system [37]. Rao et al. [104] found that intracerebellar injection of 200 µg spermine attenuated the increased production of cGMP mediated through NMDA and AMPA receptors in response to 5 µg injections of quisqualic acid and D-serine. Brundell et al. [105] proposed that intracellular spermine can bind to both the open and closed configurations of AMPARs in a voltage-independent but use-dependent manner. They found that bath application of 10 µM spermine initially potentiated and then antagonized excitatory post-synaptic currents in locust muscle in a use-dependent fashion. When injected directly into the muscle, the response was similar, except that it was not use-dependent.

Based on the observation that 40 nmol NBQX (2,3-Dihydroxy-6-nitro-7-sulfamoyl- benzo[f]quinoxaline) protected against the effects of 100 nmol of spermine, Otsuki et al. [106] suggested that AMPA receptors as well as NMDA receptors are involved in spermine-induced neurotoxicity in rat striatum. Isa et al. [107] discovered that, in rat hippocampal neurons, current responses mediated by Type II AMPARs were blocked by 170 µM spermine. These receptors have high Ca^2+^ permeability. In contrast, current responses from Type I AMPARs, which are essentially impermeable to Ca^2+^, were unaffected. EPSCs in putative non-pyramidal neurons from rat hippocampal slices mediated by Type II AMPARs were largely blocked by 1 mM spermine, while EPSCs resulting from Type I AMPARs were significantly less inhibited. Washburn and Dingledine [108] reported that spermine may bind to GluR2-lacking AMPA receptors (Type II AMPARs) at two or more distinct sites, one close to the cytoplasmic side of the ion channel and the other nearer the outer side of the channel.

Neurons bearing Type II AMPARs are especially susceptible to ischemic cell death. Noh et al. [109] reported that 1-naphthyl acetyl spermine, a selective channel blocker of Type II AMPARs, provided partial protection of rat hippocampal CA1 neurons. Motor neurons are especially vulnerable to AMPA agonists because they express Type II AMPARs [110,111,112].

#### 2.3.6. Spermine and Kainate Receptors

Kainate receptors have nonselective cation channels that are permeable to Ca^2+^ and they bind intracellular spermine very strongly so that they activate only when hyperpolarized [113]. At present, the effects of extracellular spermine on kainate receptors are unclear, owing to contradictory reports in the literature. Brackley et al. [71] found that in *Xenopus* oocytes injected with whole rat brain mRNA, responses to L-kainate were potentiated by 10–100 µM spermine. McGurk et al. [72] expressed rat brain glutamate receptors in *Xenopus* oocytes, but did not find that spermine potentiated the responses to kainate (kainate receptors) or quisqualate (AMPA receptors), as it did to NMDA. Pegg [114] opined that because kainate receptors have been linked to epilepsy-like seizure activity, modulation of kainate receptors could potentially promote seizures.

#### 2.3.7. Spermine Is a Nicotinic Acetylcholine Receptor Antagonist at µM Concentrations, but an Agonist at nM Concentrations

Anis et al. [115] reported that spermine (IC_50_ = 110 µM) inhibited the binding of both α-bungarotoxin and perhydrohistrionicotoxin to the *Torpedo* nicotinic acetylcholine receptor (nAChR) in the presence of carbamylcholine, a cholinergic agonist. Szczawinska et al. [116] likewise found that at concentrations above 1 mM, spermine inhibited ion influx and α-bungarotoxin binding to *Torpedo* electric organ nAChRs, thereby acting as a competitive antagonist of the nAChR. In contrast, at sub-µM concentrations, spermine enhanced cation influx by about 20%, effectively acting as an nAChR agonist. Using an outside-out patch and whole-cell voltage-clamp recordings from cultured *Xenopus* muscle cells, Hsu [117] obtained similar results, and concluded that the biphasic results resulted from spermine action at different sites.

Haghighi and Cooper [118] found that when spermine was added to the patch electrode in outside-out recordings, it caused a concentration- and voltage-dependent block of ACh-evoked single-channel currents. They conclude that the voltage-dependent block by intracellular spermine underlies inward rectification of neuronal nAChRs. They also found that extracellular spermine blocks both α_3_β_4_ (IC_50_ = 42.3 µM) and α_4_β_2_ receptors (IC_50_ = 40.3 µM) and suggested that extracellular spermine under pathological conditions, could selectively block these receptors. Certainly snake envenomation qualifies as a pathological condition.

On a parallel track, Law et al. [119] found that spermine inhibited the reuptake of choline by rat forebrain synaptosomes (IC_50_ = 0.22 mM). Dopamine uptake was also inhibited, but about 10-fold less potently.

#### 2.3.8. Spermine and Muscarinic Acetylcholine Receptors

Tsvilovskyy et al. [120] reported that 1 mM extracellular spermine blocks muscarinic receptor controlled Ca^2+^ currents in guinea pig ileal smooth muscle myocytes in a concentration- and voltage-dependent manner. However, because the inhibition was similar for both carbachol- and GTPgS-evoked currents, they concluded that the blockade was due to a direct action on the ion channel, rather than on the receptor itself.

#### 2.3.9. Spermine Interacts with GABAA Receptors

Intracerebroventricular injection of spermine (1.13 µmol/chick) decreased locomotion, promoted sedation or sleep, and caused a pronounced increase in GABA content of the diencephalon and brainstem [121]. Gilad et al. [122] found that spermine potentiates the binding of diazepam and flunitrazepam to rat forebrain membranes in the presence of 1 mM GABA in a concentration-dependent manner. In the presence of the non-ionic detergent, Triton X-100, larger concentrations of polyamines inhibited binding. Sabato et al. [123] and Martijena et al. [124] had previously reported enhanced binding in the presence of this detergent. Moreover, spermine inhibited binding of the peripheral-type benzodiazepine antagonist, PK 11195, at 50–500 µM concentrations, but not at concentrations above 1 mM. No effects on the binding of GABA, muscimol, or Ro 15–1788 were observed. Di Scenna et al. [24] found that mM concentrations of spermine reduced kainate, AMPA, and GABA_A_-mediated evoked potentials and concluded that their observations could best be explained by inhibition of presynaptic VDCCs.

#### 2.3.10. Spermine Sensitizes Acid-Sensing Ion Channels

Acid-sensing ion channels of subtype 1a (ASIC1a), which differ from other ASICs in that they conduct Ca^2+^ as well as Na^+^, are known to play a major role in ischemic neuronal damage [125]. Duan et al. [126] found that 250 µM extracellular spermine exacerbates ischemic neurotoxicity in mouse cortical neurons by sensitizing ASIC1a channels to extracellular acidosis. Using either a specific ASIC1a antagonist or deletion of the ASIC1 gene, ischemic neuronal damage caused by spermine, both in cultured, dissociated neurons and in a mouse ischemic model, was greatly reduced. Spermine-enhanced ASIC1a activity increases acid-induced neuronal membrane depolarization [126] and cytoplasmic Ca^2+^ overload [125], the latter being responsible for spermine-exacerbated neuronal damage. Blocked spermine synthesis reduced the damage caused by ASIC1a channels, but not that caused by NMDA receptors [126].

#### 2.3.11. Spermine Sensitizes Capsaicin Receptors

Ahern et al. [127] reported that in sensory neurons from murine nodose ganglia, 100 M spermine alone elicited no detectable current, but markedly enhanced the currents evoked by 10 nM capsaicin 2.8-fold (EC_50_ = 5 µM). In HEK293 cells, extracellular spermine activated inward currents in voltage-clamped cells. Responses to 500 M and 5 mM spermine elicited currents corresponding to 9% and 29% of that induced by 30 nM capsaicin. Gewehr et al. [128] demonstrated that nociceptive signals are transduced by capsaicin receptors (TRPV1).

#### 2.3.12. Spermine Inhibits Platelet Aggregation

Israels et al. [129] reported that spermine (14.3–47 mM) inhibited 42% to 100% of platelet aggregation induced by thrombin, arachidonic acid, and lysophosphatidic acid. It also inhibited phosphorylation of the myosin light chain induced by thrombin, but not by arachidonic acid or lysophosphatidic acid. Others later reported that spermine (1–10 mM) inhibited thrombin-induced (1.5 nM) platelet activation in a concentration-dependent manner, including 5-hydroxytryptamine release [130,131,132]. Specifically, it blocks thrombin binding to GPIb of resting platelets and fibrinogen binding to GPIIb/IIIa of activated platelets. Inhibition of platelet aggregation is completely consistent with the anticoagulant strategies of viperid and crotalid venoms [28].

#### 2.3.13. Spermine Inhibits Ca^2+^-ATPase

Spermine inhibits Ca^2+^-ATPase of skeletal muscle sarcoplasmic reticulum, in part by competing with Mg^2+^ necessary for ATPase activity [133]. Palacios et al. [134] later reported that pig brain Ca^2+^-ATPase was likewise inhibited by spermine with IC_50_s ranging from 2.5 to 27 mM, depending upon the phospholipid with which it was reconstituted. They found, as Hughes et al. also reported, that spermine does not interfere with the enzyme’s affinity for Ca^2+^ or ATP, but suggested that spermine may bind to negative charges on the enzyme. This suggests a mechanism similar to Mg^2+^ competition proposed by Hughes et al. [133], but it may also reflect an effect on lipid binding [126]. Because Ca^2+^-ATPase acts as a pump to remove Ca^2+^ from the cell, its inhibition would raise intracellular Ca^2+^ concentrations, resulting in skeletal muscle (including diaphragm) paralysis and death of all cell types [135,136]. However, it is questionable whether physiological spermine concentrations could ever approach the upper end of the sensitivity range reported by Palacios et al.

#### 2.3.14. Spermine Promotes Breakdown of the Blood–Brain Barrier

Polyamines disrupt the blood–brain barrier (BBB) under various pathological states [125,137,138,139,140]. Glantz et al. [138] reported that putrescine and spermidine, when injected into the carotid artery, caused a rapid increase in BBB permeability 1 min after injection, with a slight decline at 15 min. A slower effect was noticed after spermine administration which became significant only at 15 min. Poduslo and Curran [141] have suggested the possibility of covalently modifying proteins with polyamines in order to deliver drugs to brain areas since protein permeability increases nearly 350-fold in some cases. The relevance of this to envenomation is that exogenous spermine may permit proteinaceous venom constituents to access brain areas from which they would otherwise be excluded. However, none of the above studies measured circulating polyamine levels, so at present it is not possible to determine whether levels of polyamines induced by envenomation reach the permeability threshold of the blood–brain barrier, a threshold that may vary across species.

#### 2.3.15. Pharmacology of Spermidine, Putrescine, and Cadaverine that Is Potentially Pertinent to Envenomation

Short-chain diamines, such as putrescine and cadaverine, strongly affect the biological activity of histamine [142]. Mongar [143] found that short-chain diamines are both substrates for and inhibitors of histaminase (=diamine oxidase) and that they can potentiate the effects of histamine. Lyons et al. [144] reported that cadaverine potentiated histamine uptake in rat small intestine segments, an effect that they attributed to inhibition of histamine catabolism. This conclusion was based on the earlier work of Taylor and Lieber [145], who found that cadaverine inhibited histamine-*N*-methyltransferase and diamine oxidase, the two principal histamine catabolic enzymes in the intestine. Other workers [13,146,147,148] have also implicated putrescine in histamine potentiation. Moreover, Paik Jung and Bjeldanes [142] found that the potentiation of histamine toxicity by cadaverine is greatest when administered in conjunction with putrescine. Both histamine-*N*-methyltransferase and diamine oxidase are widely distributed among mammalian tissues [149,150]; thus it is possible that these two diamines could potentiate histamine release in envenomation, although the cadaverine titer in most venoms is probably too small to be of consequence. In support of this possibility, Marmo et al. have reported that the hypotensive effect of i.v. injections of spermine and spermidine are due to histamine release [42].

Putrescine and spermidine, as well as spermine, have been shown to potentiate transient relaxation of isolated guinea pig trachealis (smooth muscle) [43]. De Meis and De Paula specifically attributed this relaxation to inhibition of actomyosin ATPase [151].

## 3. Conclusions

Snake venoms apparently all contain polyamines, although polyamine titers in elapid venoms are manifestly minor. Cadaverine, which is produced from lysine in an anabolic pathway separate from the pathway that produces the other three polyamines, is normally the least abundant. Spermidine was absent from all six elapid venoms examined and from venom of the viperid, *Pseudocerastes fieldi*. With this exception, all other venoms contained all polyamines, although titers of individual polyamines varied by several orders of magnitude. All venoms manifested either a spermine-dominant or a putrescine dominant pattern. If venom polyamines are not sufficiently abundant to provoke systemic effects in the prey, it seems virtually certain that in the venoms of many viperids and crotalids, there are high enough polyamine concentrations to induce local effects. Endogenous polyamine stores released from prey tissues may well unleash systemic symptoms. Spermine reduces the force of cardiac contractions, and blocks vasoconstriction via blood vessel *L*-type Ca^2+^ channels. In addition, it blocks ryanodine receptors in skeletal muscle, triggers excitotoxic Ca^2+^ influx at extrasynaptic NMDA receptors, causes the death of motor neurons that bear Ca^2+^-sensitive Type 2 AMPA receptors, inhibits platelet aggregation, and breaks down the blood-brain barrier, and other effects. Putrescine, in combination with cadaverine, inhibits histamine catabolism, thereby enhancing inflammatory responses and promoting hypotension. Thus polyamine pharmacology is consistent with snake envenomation strategies to limit prey flight via hypotension and circulatory shock as well as by direct paralysis [35].

## 4. Materials and Methods

### 4.1. Venom Samples

Lyophilized crude venoms of 31 venomous snake species were examined in this study (Table B1). Venoms were dissolved in water at a concentration of 125 mg/mL. Samples of *Naja sputatrix*, *Bitis gabonica*, *Bitis nasicornis*, *Ovophis okinavensis*, and all three *Protobothrops* species were from individual snakes, as were all rattlesnake venoms supplied by the first author. All others were pooled samples from multiple individuals. Ten-microliter samples (1.25 mg) were supplied for mass spectrometry. Venoms of five prairie rattlesnakes (*Crotalus viridis viridis*) from each of two populations were examined in order to assess the amount of individual variation in polyamine levels.

### 4.2. Polyamine Derivatization

All solvents and reagents used were analytical grade and were purchased from Sigma (St. Louis, MO, USA) and ThermoFisher Scientific (Waltham, MA, USA). Freshly prepared reagents were used for polyamine derivatization. The reagents were as follows: 10% perchloric acid in water, 4% benzoyl chloride in acetonitrile, 2N sodium hydroxide in water, and saturated sodium chloride in water. Reagents were prepared in glass vials (10 mL), and all reactions were also carried out in glass vials (2 mL).

Acyl- and arylpolyamine conjugates, such as those commonly known from spider venoms, were not investigated because at present there are no published reports suggesting that snakes make them. In addition, the derivatization technique employed here cannot detect conjugated amines, if those are present. Lastly, the liquid chromatography/mass spectrometry parameters employed here were optimized only for primary polyamines. However, the presence of various other unidentified chromatographic peaks of significant intensity in each species invites further analysis.

Polyamine standards and venom polyamines were derivatized using an adaptation of the procedure of Liu et al. [9]. Stock solutions (10 mg/mL) of each polyamine standard (spermine, spermidine, putrescine, and cadaverine) were prepared in water. Stock solutions of the four individual polyamines were mixed and diluted to prepare working standard mixtures at 10, 5, and 1 mg/mL. Calibration standards (100, 50, 25, 12.5, 6.2, and 3.1 ng/150 mL) were prepared by diluting the standard mixtures with water.

To 150 mL of the calibration standard solution, 125 mL of 10% perchloric acid were added, mixed, and agitated on a shaker for 3 min. After agitation, the mixture was alkalinized with 200 mL of 2N NaOH, followed by the addition of 150 mL of 4% benzoyl chloride. This reaction mixture was mixed and sonicated for 30 min at 30–35 °C. After sonication, 500 mL of saturated NaCl solution were added and mixed well so as to react with excess benzoyl chloride. Finally, the solution was extracted twice with diethyl ether (1 mL × 2). The pooled diethyl ether extracts were dried over anhydrous MgSO_4_ (20–30 mg), filtered through a glass pipette using cotton plug, collected in a new vial, and evaporated in a Savant SpeedVac vacuum concentrator (ThermoFisher, Waltham, MA, USA). Dried derivatives were preserved at 4 °C before MS analysis.

The above mentioned standard polyamine derivatization method was used for snake venom polyamine derivatizations. Each snake venom (5 µL; concentration = 125 µg/µL) was suspended in 20 µL water (MilliQ), vortexed (30 s), and centrifuged (9000 rpm, 10 min, room temperature). An aliquot (10 µL, equivalent to 2 µL crude snake venom solution) was transferred in a glass vial (2 mL), and 140 µL of water were added, followed by the addition of 125 µL of 10% perchloric solution. The mixture was treated in a similar fashion to that used for polyamine standards and the products were extracted with ether. Dried snake venom polyamine derivatives and the polyamine standard derivatives were dissolved in 100 µL aqueous-methanol (1% water-0.01% formic acid) and analyzed by LC-MS.

### 4.3. Liquid Chromatography/Mass Spectrometry of Polyamine Standards

A Waters high-definition mass spectrometer (HDMS SYNAPT G2-S, Waters, Milford, MA, USA) was used for mass spectrometry data collection. The mass spectrometer was equipped with an ESI ion source (ZSpray) and a Waters ultra-high-pressure liquid chromatograph (ACQUITY I Class UPLC) with a Binary Solvent Manager, a Sample Manager, and a PDA eλ detector.

TOF MS spectra were generated for polyamine derivatives in positive ion mode with normal resolution at mass range m/z 100–700 Da. MS conditions were set as follows: capillary 3.0 kV, sample cone 30v, source temperature at 150 °C, desolvation temperature at 550 °C, cone gas 150 L/h, desolvation gas 900 L/h, nebulizer 6 bar, scan time 0.2 s, mass window ±0.5 Da, source offset 40. Leucine-enkephalin (m/z 120.0813 and 556.2771 with collision trap energy 18) was used as a lock spray (at 30 s intervals, 3 scans merged).

**Figure B1:**
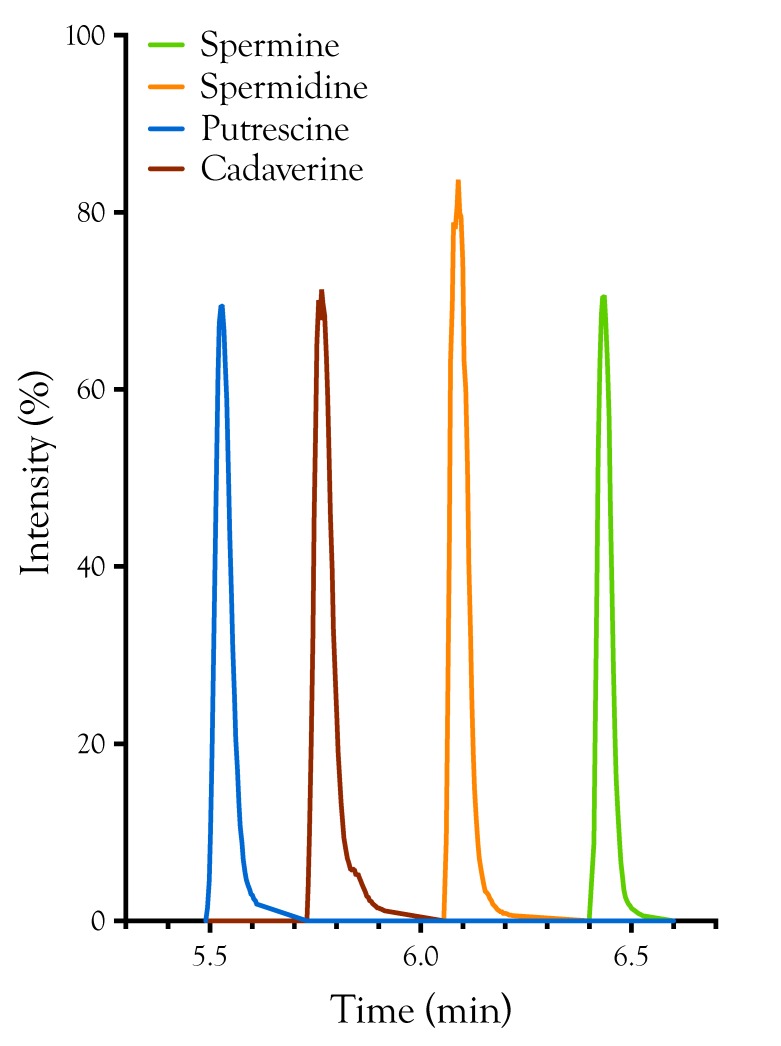
Extracted ion chromatogram (XIC) showing the elution times of the four polyamines quantified in this study.

Polyamine derivatives were separated on an Acquity BEH C_18_ column (150 × 2.1 mm, 1.7 µm, Waters). A 15-min step-gradient was used for polyamine separation (10% B for 0.0–2.0 min, 10% to 90% B for 2.0–8.0 min, hold 90% B for 8.01–10.0 min, equilibration 10% B for 10.1–15.0 min; where solvent A was water, and solvent B was acetonitrile, both solvents containing 0.1% formic acid; 300 µL/min flow rate and a column temperature of 40 °C). Samples of 3 µL were injected using the auto-sampler, and all samples were run in triplicate (Figure B1).

### 4.4. Data Processing

**Figure B2:**
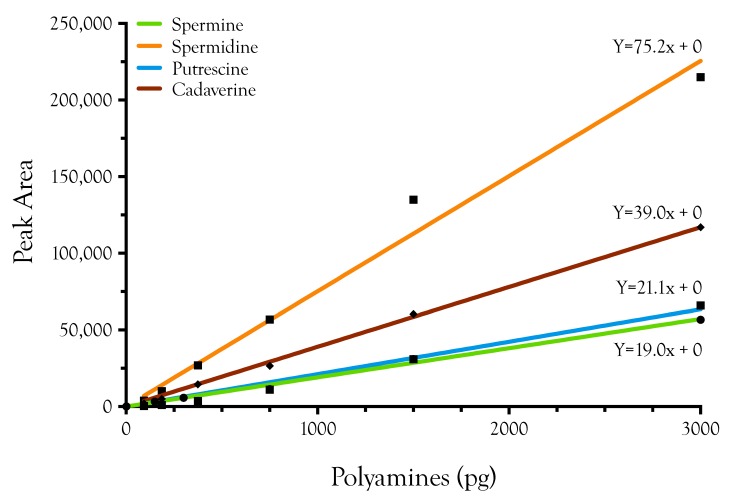
Standard curves for the four polyamines. Only two samples out of the 208 analyzed by LC-MS exceeded 3000 pg/injection. For all curves, *p* < 0.0001.

Corresponding masses for polyamine derivatives were spermine (m/z 619.3284, C_38_H_43_N_4_O_4_ MH^+^), spermidine (m/z 458.2440, C_28_H_32_N_3_O_3_ MH^+^), putrescine (m/z 297.1603, C_18_H_21_N_2_O_2_ MH^+^), and cadaverine (m/z 311.1760, C_19_H_23_N_2_O_2_ MH^+^). Peak areas for each polyamine were obtained by generating extracted ion chromatograms (XIC) using MassLynx version 4.1 (Waters, Milford, MA, USA). Polyamine concentrations in snake venom were calculated based on polyamine standard calibration curves (Figure B2).

## Figures and Tables

**Table 1:** Polyamine concentrations (µg and nmol polyamine/g venom) in venoms of 31 advanced venomous snake taxa, organized by family. Differences between taxa are large, even interspecifically, as the *Crotalus* and *Protobothrops* samples indicate. Elapid venoms are generally depauperate in polyamines. Some values contain more significant figures than are appropriate; however, this has been done to align the values so as to make the table intuitive. Full taxonomic names are provided in Apendixs.

Snake Taxa	Venom Polyamines (µg/g)					Venom Polyamines (nmol/g)				
	Spermine	Spermidine	Putrescine	Cadaverine	Total	Spermine	Spermidine	Putrescine	Cadaverine	Total
**Elapidae**										
*B. multicinctus*	0.006	0.000	0.083	0.038	0.128	0.032	0.000	0.944	0.376	1
*D. polylepis*	0.006	0.000	0.064	0.013	0.083	0.032	0.000	0.726	0.125	1
*M. surinamensis*	0.006	0.000	0.064	0.013	0.083	0.032	0.000	0.726	0.125	1
*N. kaouthia*	0.006	0.000	0.064	0.013	0.083	0.032	0.000	0.726	0.125	1
*N. sputatrix*	0.006	0.000	0.064	0.013	0.083	0.032	0.000	0.726	0.125	1
*O. hannah*	0.013	0.000	0.077	0.019	0.109	0.063	0.000	0.871	0.188	1
**Mean**	**0.007**	**0.000**	**0.069**	**0.018**	**0.095**	**0.037**	**0.000**	**0.787**	**0.177**	**1**
**Std. Deviation**	**0.003**	**0.000**	**0.009**	**0.010**	**0.019**	**0.013**	**0.000**	**0.097**	**0.100**	**0**
**Viperidae**										
*B. gabonica*	1.178	0.166	3.085	0.166	4.595	5.820	1.146	34.995	1.628	44
*B. nasicornis*	81.158	3.104	12.864	1.568	98.694	401.099	21.370	145.933	15.345	584
*C. cerastes*	246.477	9.971	38.003	2.899	297.350	1218.132	68.649	431.117	28.373	1746
*D. palestinae*	2.835	0.160	6.259	1.504	10.758	14.012	1.102	71.006	14.719	101
*D. r. siamensis*	50.816	2.080	35.866	1.555	90.317	251.142	14.320	406.870	15.220	688
*P. fieldi*	0.019	0.000	0.102	0.013	0.134	0.095	0.000	1.162	0.125	1
**Mean**	**63.747**	**2.580**	**16.030**	**1.284**	**83.642**	**315.050**	**17.764**	**181.847**	**12.569**	**527**
**Std. Deviation**	**95.443**	**3.834**	**16.751**	**1.066**	**113.557**	**471.697**	**26.395**	**190.031**	**10.428**	**664**
**Crotalidae**										
*A. c. contortrix*	220.019	0.448	6.381	1.523	228.371	1087.347	3.084	72.386	14.907	1178
*A. p. leucostoma*	80.627	0.627	1.280	0.307	82.842	398.474	4.318	14.521	3.006	420
*A. nummifer*	0.051	0.006	0.077	0.013	0.147	0.253	0.044	0.871	0.125	1
*B. erythromelas*	0.058	0.006	0.064	0.013	0.141	0.285	0.044	0.726	0.125	1
*B. moojeni*	0.608	0.083	0.493	0.102	1.286	3.005	0.573	5.590	1.002	10
*B. schlegelii*	104.467	1.466	6.374	1.504	113.811	516.295	10.090	72.313	14.719	613
*C. rhodostoma*	67.962	0.154	6.227	1.498	75.840	335.878	1.057	70.643	14.656	422
*C. sasai*	0.160	0.006	0.077	0.013	0.256	0.791	0.044	0.871	0.125	2
*C. adamanteus*	10.822	2.688	11.712	1.568	26.790	53.486	18.506	132.864	15.345	220
*C. cerastes*	3.885	2.144	7.974	1.510	15.514	19.199	14.761	90.464	14.782	139
*C. d. terrificus*	30.598	12.890	23.725	1.670	68.883	151.223	88.741	269.141	16.348	525
*C. m. pyrrhus*	0.115	0.013	0.122	0.013	0.262	0.569	0.088	1.379	0.125	2
*C. v. concolor*	1.984	1.062	1.216	0.019	4.282	9.805	7.314	13.795	0.188	31
*C. v. viridis*	2.972	1.238	1.317	0.022	5.549	14.687	8.524	14.942	0.216	38
*L. stenophrys*	26.240	0.902	2.022	0.301	29.466	129.683	6.213	22.943	2.944	162
*O. okinavensis*	0.090	0.006	0.070	0.019	0.186	0.443	0.044	0.799	0.188	1
*P. elegans*	469.459	0.179	6.214	1.504	477.357	2320.150	1.234	70.498	14.719	2407
*P. flavoviridis*	17.376	0.173	6.208	1.498	25.254	85.875	1.190	70.425	14.656	172
*P. mucrosquamatus*	1602.214	11.315	61.747	14.963	1690.240	7918.426	77.902	700.479	146.440	8843
**Mean**	**138.932**	**1.864**	**7.542**	**1.477**	**149.815**	**686.626**	**12.830**	**85.561**	**14.454**	**799**
**Std. Deviation**	**372.175**	**3.699**	**14.341**	**3.346**	**390.393**	**1839.355**	**25.468**	**162.691**	**32.745**	**2033**

## Appendix A

**Table A1:** Polyamine measurements in venoms of 10 *Crotalus viridis viridis* from two populations in western Colorado (nmol polyamine/g venom). Two replicates were run for each specimen and each polyamine.

Specimen	Spermine [nmol/g]		Spermidine [nmol/g]		Putrescine [nmol/g]		Cadaverine [nmol/g]		Total
C. v. viridis 1501	24.640	24.576	11.280	11.324	28.896	29.695	0.251	0.251	65.456
C. v. viridis 1502	17.776	17.586	9.561	9.385	9.511	9.366	0.188	0.188	36.781
C. v. viridis 1505	17.555	16.985	10.355	10.134	29.913	30.130	0.376	0.376	57.912
C. v. viridis 1506	8.761	8.445	6.565	6.565	18.296	18.223	0.188	0.251	33.648
C. v. viridis 1507	15.277	17.302	11.015	11.720	12.996	12.923	0.188	0.188	40.805
C. v. viridis 301	14.993	15.562	7.182	7.667	15.174	15.392	0.188	0.188	38.173
C. v. viridis 304	12.873	13.000	9.694	9.253	10.745	11.326	0.188	0.188	33.634
C. v. viridis 306	9.932	9.647	7.050	6.962	13.359	12.778	0.188	0.188	30.052
C. v. viridis 308	16.036	16.131	6.213	6.169	3.412	3.340	0.188	0.188	25.838
C. v. viridis 311	8.287	8.382	6.257	6.125	6.680	6.680	0.188	0.188	21.393
**Mean**	**14.613**	**14.762**	**8.517**	**8.530**	**14.898**	**14.985**	**0.213**	**0.219**	**38.369**
**Std. Deviation**	**4.955**	**5.034**	**2.052**	**2.114**	**8.722**	**8.914**	**0.061**	**0.061**	**13.676**

**Table A2:** Polyamine abundances (nmol polyamine/g venom) in relation to preferred prey of adult snakes.

Taxon	Polyamines (nmol/g venom)				Prey Species							References
	Spermine	Spermidine	Putrescine	Cadaverine	Arthropods	Fish	Birds	Lizards	Anurans	Snakes	Mammals	
**Elapidae**												
*B. multicinctus*	0.032	0.000	0.944	0.376	-	❋	-	-	-	❋	-	Mao, 1970 [152]
*D. polylepis*	0.032	0.000	0.726	0.125	-	-	❋	-	-	-	❋	Branch et al., 1995 [153]
*M. surinamensis*	0.032	0.000	0.726	0.125	-	❋	-	-	-	-	-	Olamendi-Portugal et al., 2008 [154]; Morais et al., 2011 [155]
*N. kaouthia*	0.032	0.000	0.726	0.125	-	-	-	-	-	❋	❋	Chanhome et al., 2001 [156]; Modal et al., 2016 [157]
*N. sputatrix*	0.032	0.000	0.726	0.125	-	-	-	-	-	❋	❋	Inferred
*O. hannah*	0.063	0.000	0.871	0.188	-	-	-	❋	-	❋	❋	Chanhome et al., 2001 [156]
**Mean**	**0.037**	**0.000**	**0.787**	**0.177**								
**Std. Deviation**	**0.013**	**0.000**	**0.097**	**0.100**								
**Viperidae**												
*B. gabonica*	5.820	1.146	34.995	1.628	-	-	❋	❋	❋	-	❋	Luiselli & Akani, 2003 [158]
*B. nasicornis*	401.099	21.370	145.933	15.345	-	-	-	-	❋	-	❋	Luiselli & Akani, 2003 [158]
*C. cerastes*	1218.132	68.649	431.120	28.373	-	-	-	❋	-	-	-	Bazaa et al., 2005 [159]
*D. palestinae*	14.012	1.102	71.006	14.719	-	-	-	-	-	-	❋	Inferred
*D.siamensis*	251.142	14.320	406.870	15.220	-	-	-	❋	❋	❋	❋	Chanhome et al., 2001 [156]; Gennaro et al., 2007 [160]
*P. fieldi*	0.095	0.000	1.162	0.125	-	-	❋	❋	-	-	❋	Ali et al., 2015 [161]
**Mean**	**315.050**	**17.764**	**181.848**	**12.569**								
**Std. Deviation**	**471.697**	**26.395**	**190.032**	**10.428**								
**Crotalidae**												
*A. c. contortrix*	1087.374	3.084	72.386	14.907	❋	-	❋	❋	❋	❋	❋	Trauth & McAllister, 1995 [162]; Heatwole et al., 1999 [163]
*A. p. leucostoma*	398.474	4.318	14.521	3.006	-	❋	-	-	❋	❋	❋	Himes, 2003 [164]; Vincent et al., 2004 [165]
*A. nummifer*	0.253	0.044	0.871	0.125	-	-	-	-	-	-	❋	Martins et al., 2002 [166]
*B. schlegelii*	0.285	0.044	0.726	0.125	-	-	❋	❋	❋	-	❋	Greene, 1988 [167]; Sorrel, 2007 [168]
*B. erythromelas*	3.005	0.573	5.590	1.002	❋	-	❋	-	❋	-	❋	Martins et al, 2002 [166]
*B. moojeni*	516.295	10.090	72.313	14.719	❋	-	❋	❋	❋	❋	❋	Nogueira et al., 2003 [169]
*C. rhodostoma*	335.878	1.057	70.643	14.656	❋	❋	❋	❋	❋	❋	❋	Daltry et al., 1998 [156]; Chanhome et al., 2001 [170]
*C. sasai*	0.791	0.044	0.871	0.125	❋	-	-	❋	❋	-	❋	Luiselli, 2006 [166]; Martins et al., 2002 [171]
*C. adamanteus*	53.486	18.506	132.864	15.345	-	-	-	-	-	-	❋	Margres et al., 2015 [172]
*C. cerastes*	19.199	14.761	90.464	14.782	-	-	-	❋	-	-	❋	Secure & Nagy, 1994 [173,174]
*C. d. terrificus*	151.223	88.741	269.141	16.348	-	-	-	-	-	-	❋	Salomao et al, 1995 [160]; Sant’anna & Abe, 2007 [175]; Gennaro et al., 2007 [176]
*C. m. pyrrhus*	0.569	0.088	1.379	0.125	-	-	-	-	-	-	❋	Meik et al., 2012 [177]
*C.v.concolor*	9.805	7.314	13.795	0.188	-	-	-	❋	-	-	❋	Mackessy et al., 2003 [178]
*C. v. viridis*	14.687	8.524	14.942	0.216	-	-	❋	❋	-	-	❋	Hayes, 1992 [179]
*L. stenophrys*	129.683	6.213	22.943	2.944	-	-	-	-	-	-	❋	Voss, 2013 [180]
*O. okinavensis*	0.443	0.044	0.799	0.188	-	-	❋	❋	❋	-	❋	Mori & Toda, 2011 [181]
*P. elegans*	2320.150	1.234	70.498	14.719	-	-	-	❋	-	-	❋	SDA, pers. observations
*P. flavoviridis*	85.875	1.190	70.425	14.656	-	-	❋	-	❋	-	❋	Gennaro et al., 2007 [160]; Chijiwo et al., 2003 [182]
*P. mucrosquamatus*	7918.426	77.902	700.479	146.440	-	-	❋	-	❋	❋	❋	Mao, 1970 [152]
**Mean**	**686.626**	**12.830**	**85.561**	**14.454**								
**Std. Deviation**	**1938.355**	**25.468**	**162.691**	**32.745**								

## Appendix B

**Table B1:** Venomous snake taxa examined in this study and geographic origins of samples. Samples from Zooherp and SDA were obtained from single snakes. All other samples were pooled from multiple individuals. Sources: Biotoxins, St. Cloud, FL, USA; Clodomiro Picado, San Juan, Costa Rica; CEPB, Centro de Estudos e Pesquisas Biológicas, Pontifícia Universidade Católica de Goiás, Goiânia, Goiás, Brasil; KRZ, Kentucky Reptile Zoo, Slade, KY, USA; SVRI, Snake Venom Research Institute, Nanning, China; Zooherp, Salt Lake City, UT, USA.

Family	Taxon	Origin	Source
Elapidae	*Bungarus multicinctus*	China	SVRI
	*Dendroaspis polylepis*	Kenya	Biotoxins
	*Micrurus surinamensis*	Letícia, Colombia	CEPB
	*Naja kaouthia*	Unknown	KRZ
	*Naja sputatrix*	Java	Zooherp
	*Ophiophagus hannah*	Unknown	KRZ
Viperidae	*Bitis gabonica*	Burundi	Zooherp
	*Bitis nasicornis*	Unknown	Zooherp
	*Cerastes cerastes*	Unknown	KRZ
	*Daboia palestinae*	Unknown	KRZ
	*Daboia russellii siamensis*	Thailand	SVRI
	*Pseudocerastes fieldi*	Unknown	KRZ
Crotalidae	*Agkistrodon contortrix contortrix*	South Carolina	Biotoxins
	*Agkistrodon piscivorus leucostoma*	Missouri	KRZ
	*Atropoides nummifer*	SE Mexico	KRZ
	*Bothriechis schlegelii*	Costa Rica	Clodomiro Picado
	*Bothrops moojeni*	Goiás, Brasil	CEPB
	*Bothrops erythromelas*	NE Brazil	KRZ
	*Calloselasma rhodostoma*	Thailand	KRZ
	*Cerrophidion sasai*	Costa Rica	Clodomiro Picado
	*Crotalus adamanteus*	Florida	Biotoxins
	*Crotalus durissus terrificus*	Brazil	CEPB
	*Crotalus cerastes*	Yuma, Arizona	SDA
	*Crotalus mitchellii pyrrhus*	Yavapai, Arizona	SDA
	*Crotalus viridis concolor*	Rio Blanco, Colorado	SDA
	*Crotalus viridis viridis*	Rio Blanco, Colorado	SDA
	*Lachesis stenophrys*	Costa Rica	Clodomiro Picado
	*Ovophis okinavensis*	Okinawa	SDA
	*Protobothrops elegans*	Itoman, Okinawa	SDA
	*Protobothrops flavoviridis*	Itoman, Okinawa	SDA
	*Protobothrops mucrosquamatus*	Nago, Okinawa	SDA
